# Assisting during microsurgery: tips for success

**Published:** 2023-12-01

**Authors:** Jacqueline Newton

**Affiliations:** 1 Staff Nurse: Flying Eye Hospital, Orbis International, Cape Town, South Africa.

**Knowing how to handle instruments and sharps safely, and pass them correctly during surgery, are some of the key skills scrub nurses/technicians must learn.**

**Figure:**
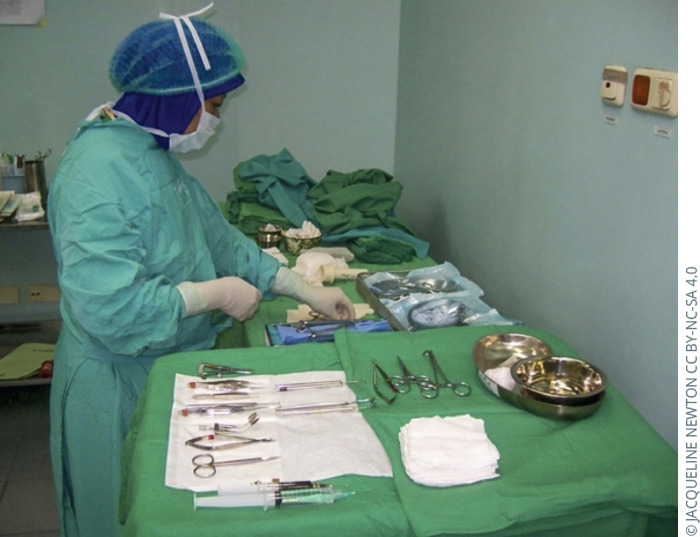
A nurse sets up the sterile field in preparation for surgery. **INDONESIA**

Simulation training is very helpful in practicing to pass instruments safely. Nurses can take turns to role play as the surgeon and scrub nurse/technician, and can experience what it is like for the surgeon to perform surgery while looking through the microscope or using magnifying loupes. These tips are equally useful for simulation training and surgical procedures.

## Handling sharps safely

**Figure 1:**
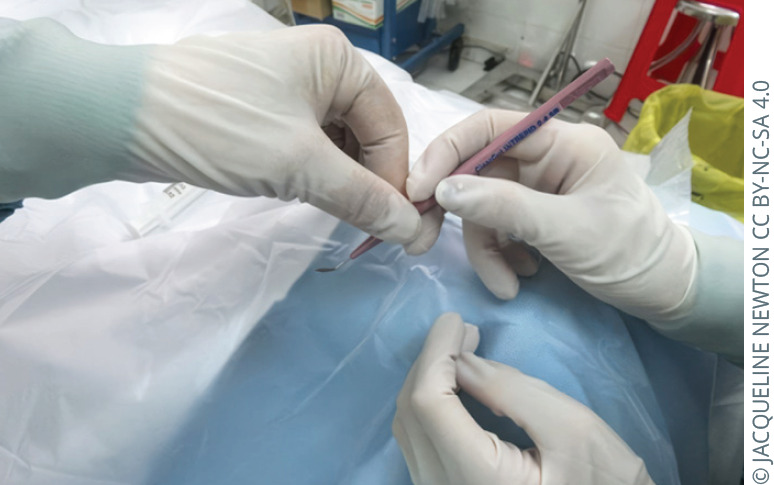
Passing a keratome blade to the surgeon with the sharp end facing away from them.

**Figure 4:**
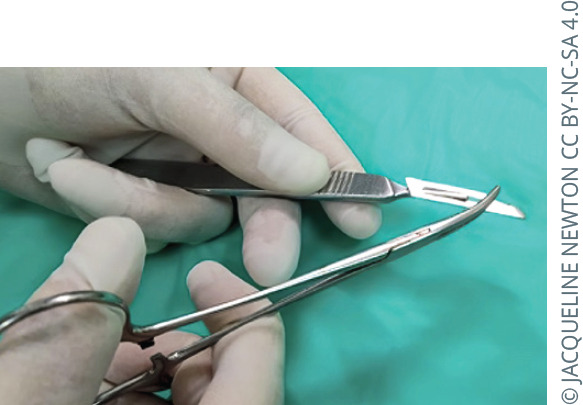
Using a clamp to remove a blade from the Bard Parker handle.

1. Hand over sharp instruments to the surgeon with the sharp end facing away from them (Figure 1). The surgeon may reach out their hand towards the sterile field and injure themselves if the sharp end is facing towards them.
2. When loading and removing blades from the Bard Parker blade handle, use a clamp (not your fingers), with the point of the blade facing in a downward position, away from yourself (Figure 4).
3. Never recap a needle. If you need to reuse a syringe, remove the needle from the syringe and replace it with a new needle. Remember: cystotome needles are also considered a sharp.
4. Be aware that a phacoemulsification tip/needle is sharp and should be covered by the plastic test chamber when not in use. It must be removed from the phacoemulsification handpiece before sending it for reprocessing.

**Before you start**

**Figure 2:**
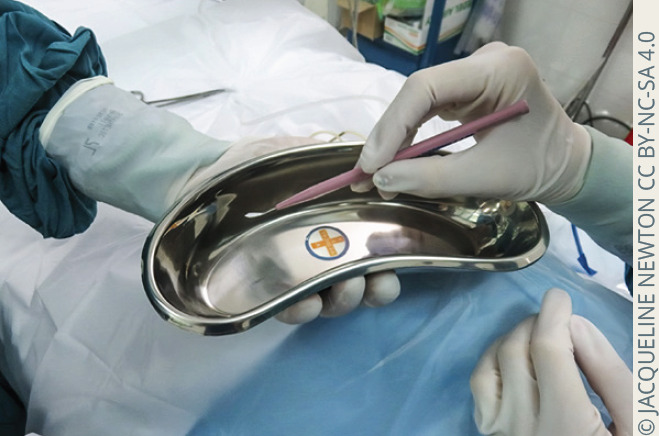
Surgeon placing sharp in a receiver held by the surgical nurse or technician.

**Figure 3:**
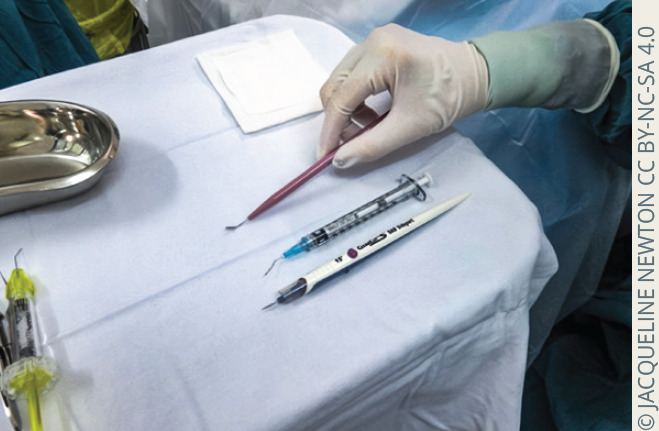
Surgeon returning a blade to the neutral zone on the sterile field.

- **Ensure the surgeon is comfortable.** Before surgeons begin their pre-surgical handwash and don a sterile gown and gloves, ask them to check the position of the operating microscope in relation to the operating room table and surgical chair being used, and to check the position of the foot switches to ensure they are comfortable.
- **Ensure the patient is comfortable.** A comfortable patient is better able to cooperate during eye surgery. To keep patients comfortable during cataract surgery (if the eye drape doesn't have a fluid collecting bag), place a gauze swab at the side of the head to absorb fluid and prevent it from running into the patient's ear.
- **Find out whether the surgeon is right- or left-handed.** This will determine how you load sutures and pass instruments.
- **Agree how the surgeon will pass sharps back to the scrub nurse/technician after use.** The surgeon can say, “Sharp back” and place the used sharp in a receiver provided by the scrub nurse/technician (Figure 2), or place the sharp on the sterile field in an area designated only for sharps, known as the ‘neutral zone’ (Figure 3).

## Passing instruments

**Figure 5:**
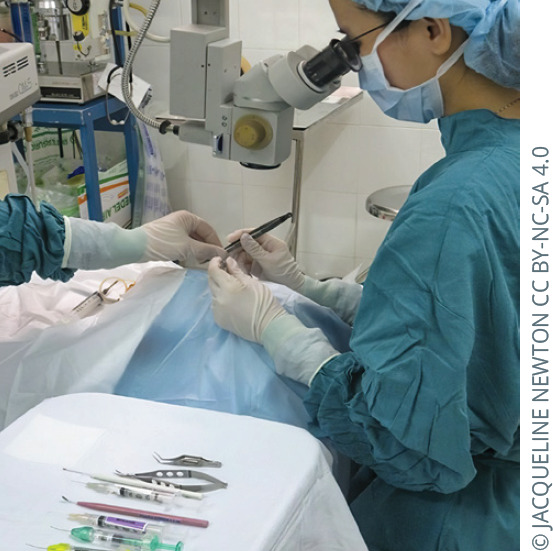
The scrub nurse/technician is positioned diagonally across from the surgeon. Passing instruments correctly allows the surgeon to work without having to stop looking through the microscope.

**Figure 6:**
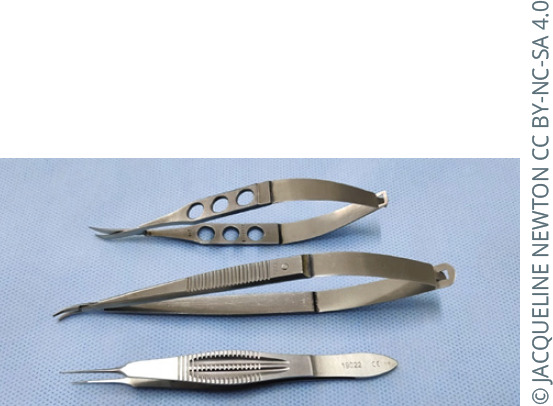
The grip section on the instrument handle has ridges or holes that allow surgeons to grasp them securely. **VIETNAM**

**Figure 7:**
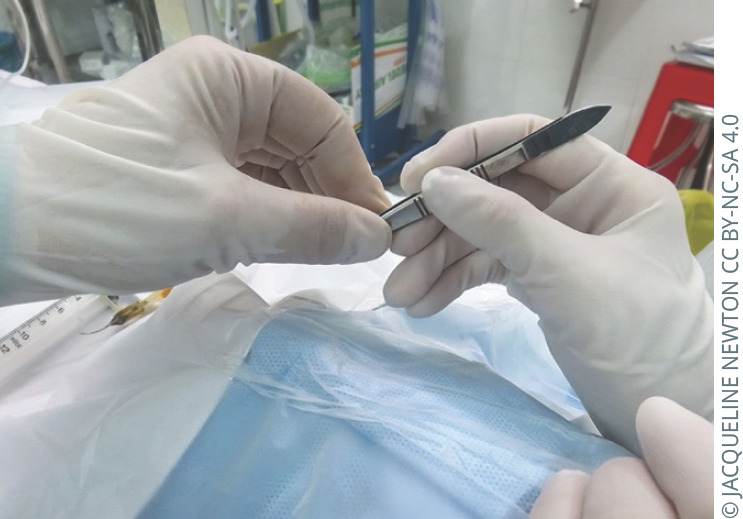
The scrub nurse/technicial (left) passes the instrument to the surgeon (right). The scrub nurse/technician's fingers do not obscure the grip section of the instrument, which allows the surgeon to grasp the instrument in the correct position. **VIETNAM**

1. When assisting the surgeon, it is good practice for the scrub nurse or technician to position themselves diagonally across from the surgeon, allowing them to pass instruments safely and comfortably (Figure 5).
2. All surgical instruments have a section on the instrument handle where the surgeon's fingers will hold or grasp the instrument (Figure 6). When passing an instrument to the surgeon, the scrub nurse/tech must ensure that their fingers do not obscure this area, as this would make it difficult for the surgeon to grip the instrument (Figure 7).
3. Place the instrument in the hands of the surgeon at the correct angle and position (as if they are using a pen or pencil), so that the instrument can be used immediately, without the need to adjust it.
4. When the surgeon is looking through the microscope, pass the instrument so that the grip section of the instrument touches either the thumb and/or index finger; this allows the surgeon to feel when the instrument can be grasped. A surgeon who is using magnifying loupes, e.g., during strabismus or oculoplastic surgery, will have limited peripheral vision and will also rely on the scrub nurse/technician to place the instruments in their hands, ready for use.
5. Remember to make appropriate adjustments for left-handed surgeons.

## Other best practices and considerations when assisting a surgeon during surgery

**Figure 8:**
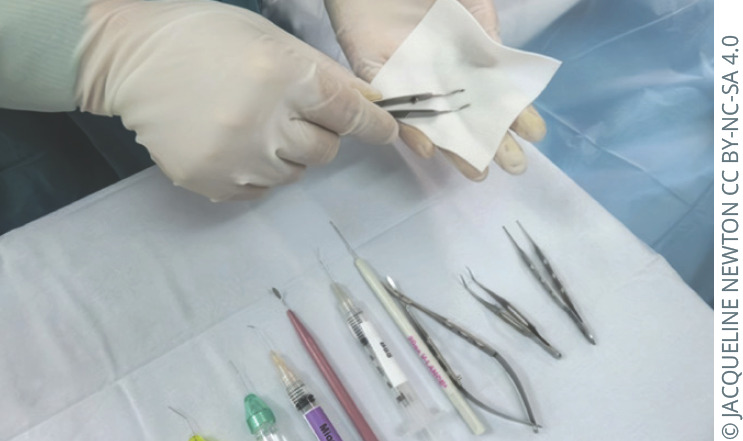
The scrub nurse/technician wipes surgical instruments after use with a soft lint-free wipe. **VIETNAM**

1. All medications drawn up and on the sterile field need to be labeled. Tell the surgeon the type of medication and dose when passing it to them.
2. All instruments which have been used during the administration of a cytotoxic agent, such as mitomycin C or 5-FU, must be placed in an area separate from the instruments that will be used to complete the operation. At the end of the procedure, these instruments can be rinsed with sterile water. Reprocessing is the same as for other instruments. The blades, needles and pledgetts used during the surgery must be placed in a sharps container labeled ‘Cytotoxic.’
3. Wipe instruments immediately after use using a soft lint-free cloth. Blood, debris and viscoelastic should not be allowed to dry on the instruments (Figure 8).
4. When loading a suture, ensure you use the tip of the needle holder to grip the needle. Avoid loading the suture close to the sharp point or swage (the connection point of the suture and the needle). See www.cehjournal.org/article/passing-sutures.
5. If a suture is missing at the time of the WHO Safe Surgery Sign Out (see panel), the surgeon must inspect the wound, confirm that the suture needle is not in the wound, and sign the intraoperative notes.
6. There are different methods for loading intraocular lenses, depending on the brand and packaging. Before loading the lens, flush the optic with balanced salt solution (BSS) and fill the cartridge with viscoelastic. Avoid using fingertips to touch the intraocular lens.
7. Always have gauze, micro sponges and/or cotton tips readily available, and in reach of the surgeon, for soaking up blood or fluids from the wound.
8. All cannulated instruments and cannulas should be primed before use to prevent air bubbles entering the eye. When priming cannulas, hold your hand or a gauze swab around the tip of the cannula to prevent the solution from being sprayed into the air or into the eyes of a surgical team member. Using Luer lock syringes will prevent cannulas and needles from shooting off the syringe when under pressure.
9. Use sterile water to rinse or wipe instruments during surgery and as soon as the surgeon returns the instrument to the neutral zone or container. **Do not use saline or balanced salt solution (BSS) to rinse instruments, as this is harmful to the instruments.** At the end of the procedure, flush cannulated instruments with sterile water on the surgical field before sending them for reprocessing.
10. Remove any damaged instruments and set them aside in a designated area. This will avoid the frustration of a damaged instrument being reprocessed and ending up back in the instrument tray for another operation.

## Complete the World Health Organization (WHO) Safe Surgery ‘Time Out’ checklist.

The surgical team must stop and check the following before starting the operation:

- The type of implant, if applicable (if an intraocular lens is planned, confirm the specific power of the lens, whether it is in the operating theatre, and whether a spare is available)
- The specific equipment needed
- Whether the instruments are sterile
- An initial count of the number of surgical instruments and consumables on the surgical field, including: suture needles, sharps (blades, hypodermic needles), retractors, pledgetts (used in glaucoma surgery), trocars and scleral plugs (used in retinal surgery), and sponges or gauze (used in oculoplastic surgery)
- Any anticipated issues or concerns that may arise during surgery, and what equipment, instruments and consumables must be available on standby
- Has the surgeon notified the surgical team of any non-routine steps?

After surgery, a final count of all the surgical instruments and consumables needs to be done for the ‘Sign Out’ section of the WHO Safe Site Surgery Checklist. The count needs to be confirmed with the surgical team and any discrepancies must be documented in the intraoperative notes.

**Table 1:**
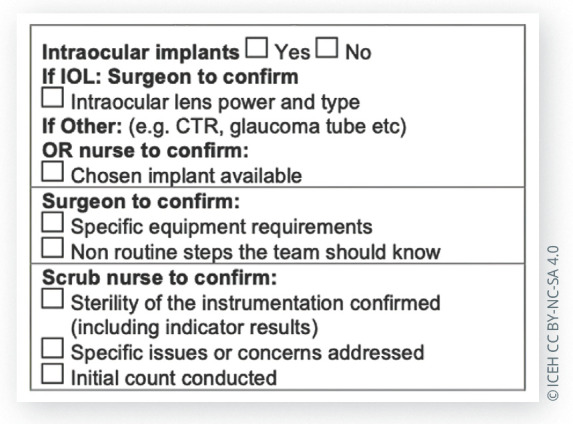
The World Health Organization ‘Time Out’ checklist, adapted for eye surgery.

**Table 2:**
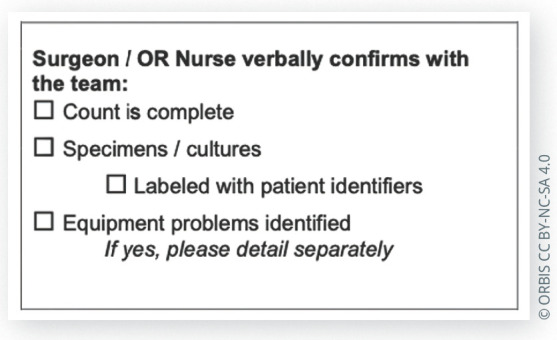
The ‘Sign Out’ section used on the Orbis Flying Eye Hospital.

